# Valuing the individual – evaluating the Dignity Care Intervention

**DOI:** 10.1177/09697330221122902

**Published:** 2022-10-07

**Authors:** Annika Söderman, Carina Werkander Harstäde, Maria Hälleberg Nyman, Karin Blomberg

**Affiliations:** Faculty of Medicine and Health, School of Health Sciences, 6233 Örebro University, Örebro, Sweden; Centre for Collaborative Palliative Care, Department of Health and Caring Sciences, Faculty of Health and Life Sciences, Linnaeus University, Växjö, Sweden; Faculty of Medicine and Health, School of Health Sciences, Örebro University, Örebro, Sweden; Department of Orthopaedics, Faculty of Medicine and Health, 6233 Örebro University, Örebro, Sweden; Faculty of Medicine and Health, School of Health Sciences, 6233 Örebro University, Örebro, Sweden

**Keywords:** dignity, intervention, mixed method, older persons, palliative care

## Abstract

**Background:**

Palliative care needs in older persons can endanger their dignity. To provide dignity-conserving care to older persons, the Swedish Dignity Care Intervention (DCI-SWE) can be used. The DCI-SWE is built on Chochinov’s dignity model and the original version, developed and tested in UK and Scotland.

**Aim:**

To describe older persons’ and their relatives’ experiences of dignity and dignity-conserving care when using the DCI-SWE in municipal health care.

**Research design:**

A mixed method study with convergent parallel design.

**Participants and research context:**

The DCI-SWE was used and evaluated in a Swedish municipality health care context. Older persons’ (*n*=17) dignity-related distress and quality of life were assessed after the intervention. Interviews with older persons (*n*=10) and their relatives (*n*=8) were analysed using thematic analysis.

**Ethical considerations:**

The study followed the World Medical Association Declaration of Helsinki. Ethical approval was obtained from the Regional Ethical Review Board in Uppsala, Sweden (Reg No. 2014/312) and the National Swedish Ethical Review Authority (Reg. No. Ö 10-2019). Informed consent was collected from older persons and their relatives.

**Findings:**

The older persons’ dignity-related distress did not significantly change over time (*p =* 0.44) neither was their overall quality of life (*p* = .64). Only psychological quality of life was decreased significantly (*p =* 0.01). The older persons and their relatives emphasized the importance of valuing the individual.

**Conclusions:**

The DCI-SWE provides a forum to talk about dignity issues, but relevant competence, continuity and resources are needed. Psychological care actions and health care professionals’ communication skills training are important. To fully evaluate, the DCI-SWE a larger sample and validated instruments are necessary.

## Introduction

Palliative care intends to support persons to live as fully as possible until they die.^1^ Of the estimated global number of persons needing palliative care at the end of life (20.4 million), 94% are adults, 69% of whom are over 60 years old,^2^ making it relevant to emphasize this care approach for older persons as they move towards the end of life, often implicating palliative care needs.

With ageing, natural changes and challenges cannot be avoided. Often older persons endure frailty, defined as ‘an increased risk of chronic disease, loss of independence, mortality and increased health care use’.^3^ Psychosocial support is required to help a person accept the situation and maintain a sense of dignity.^4^ Therefore, this study put emphasis on dignity-conserving care for older persons.

### Background

It has earlier been reported that about 50% of older persons at nursing homes experience that their dignity is affected.^5^ Both older persons and their families have described the importance of quality of life and death with dignity in palliative care, and their wish for supporting care models including these aspects.^6^

Adequate palliative care can relieve dignity-related distress and ease the suffering.^7^ A person receiving health care has dignity when able to live in accordance with their standards and values.^8^ To prevent older persons’ dignity being affected, Healthcare Professionals (HCPs) must consider their needs and observe and handle concerns.^9^ Dignity-conserving approaches can help older persons feel respected^10^ and HCPs can be guided in providing relevant palliative care.^11^

Dignity interventions are rare,^12,13^ and more research is needed to make them a solid part of older persons’ health care. An integrated review^12^ showed that dignity-conserving care (DCC) is often evaluated based on outcomes connected to illness-related concerns, but rarely based on dignity or social outcomes.

### The Dignity Care Intervention

The Dignity Care Intervention (DCI) was developed in the UK to maintain dignity in persons with palliative care needs. It intends to give clinical guidance on DCC based on a conversation support for community nurses (CNs) and suggested care actions.^10,14,15,16^ The DCI is based on Chochinov’s dignity model^17^ and includes three parts that are used in a process: the Patient Dignity Inventory (PDI) with 26 items,^18,19^ reflective questions and evidence-based care actions related to dignity. All items, reflective questions or care actions are related to one of the three main themes as follows: illness related concerns, dignity-conserving repertoire or social dignity inventory. The DCI has been translated and adapted into Swedish – ‘DCI-SWE’.^18,20,21^ In a previous study,^22^ the DCI-SWE was tested in home health care where CNs reported it gave structure to palliative care and affirmed older persons. In the UK, it has been evaluated from patients’^15^, but not from relatives´ perspectives. To enable DCC, further studies are needed. Therefore, this study aimed to describe older persons’ and their relatives’ experiences of dignity and DCC when using the DCI-SWE in municipal health care.

## Methods

### Research design

This mixed method study was guided by the Medical Research Council’s guidelines.^23^ A convergent parallel design^24^ was used, which meant different data were equally prioritized, collected in parallel and analysed separately allowing for both convergent and complementary results.^25^ By using mixed methods, this research became more rigorous and a better understanding of the DCI-SWE was enabled.^26,27,28^

### Procedure using the intervention

During two workshops (2 hours each), 28 CNs got information and training on the DCI-SWE. They were instructed to use the DCI-SWE process (described in Söderman et al., 2021) with three to five older persons each. The process starts with asking the older persons if they accept the use of the intervention in their care. Then, the older person fills in the PDI (26 items rated on a Likert scale, range 1–5), alone or with help from the CN. Those items rated by the person as ≥ 3 the CN then continues with and use the reflective questions to enhance the knowledge of the persons’ needs and concerns. Further, a discussion on care actions related to the concern in question is held by the CN together with the older person to plan the forthcoming health care. The individual time for the DCI-process depend on how many conversations are performed and what care actions are planned.

### Research context and participants

The study context was one urban and rural home health care unit, and two urban nursing homes in one large municipality in the middle of Sweden. All units provided palliative care (for further description, see Table 1). The CNs at the health care units were invited by researchers and first line managers to participate in the study and thus, use the DCI with at least three older persons each. It was planned for 20 CNs to participate in the study; hence, the researchers trained 28 CNs in the workshops, but only 11 used the DCI-SWE with older persons. This due to lack of resources during the pandemic COVID-19 and sufficient engagement.

**Table 1.** Included nursing homes and home health care units, along with an overview of the data collection.

Type of unit	Type of environment	Description of unit	Health care available	Number of older persons invited to participate	Number of older persons included	Number of baselines	Number of follow-ups	Number of interviews with older persons	Number of interviews with relatives
**Nursing home A**	Urban	71 apartments distributed over eight units. Four units are care residences, one is a group residence for persons with dementia, one is a care residence for older persons with dementia, and two are departments for older persons with mental disabilities Seven nurses were employed; two nurses used the DCI-SWE.	One nurse is available during daytime and evenings. At night, a nurse patrol service is available. The older persons have a nurse in charge of their care, and a physician can visit their apartments if needed. An occupational therapist and physiotherapist can be contacted if needed	16	2	2	2	1	1
**Home care**	Urban and rural	About 500 older persons receiving home health care 19 district nurses/nurses are employed; three district nurses/nurses used the DCI-SWE.	The efforts are carried out up to the level of nurse assistants, nurses and occupational therapists. A nurse is available 24 hours a day; the physiotherapist is available during daytime. Occupational therapists are available upon appointment. The physicians and emergency health care are the responsibility of the county council	16	10	7	7	4	3
**Nursing home B**	Urban	81 apartments distributed over ten units. Three units are for persons with dementia, while seven units have a somatic focus Five district nurses/nurses were *permanently* employed; six district nurses/nurses used the DCI-SWE.	The unit is staffed with nursing staff around the clock. Community nurses are available at daytime and in the evenings, and an occupational therapist is available during daytime on weekdays. At night, a nurse patrol service is available. The unit works in collaboration with a health care centre and through this has access to physicians and physiotherapists	13	11	9	8	5	4
In total (all healthcare units)				45	23 (51%)	18 (40%)	17 (38%)	10 (22%)	8

Older persons were identified by CNs using inclusion and exclusion criteria (Table 2), and who asked them whether the researcher could contact them. The surprise question,^29^ ‘Would I be surprised if this person died within a year?’ was used if CNs struggled to identify older persons for the study. If the answer was ‘No’ this indicated that the person had palliative care needs and could be included. One researcher (A.S.) provided older persons with further information about the study. Out of a consecutive sample of 45 older persons invited to participate, 23 agreed to take part in the study and were included. Almost all had problems with hearing and sight, although all could hear and understand verbal conversations. At baseline (when the intervention started), the sample decreased to 18 as two older persons could not attend because of COVID-19; one was discovered having severe memory problems, one died and one dropped out because of lack of interest. At follow-up, another older person had died, leading to a sample of 17 older persons. Of these persons, seven had home care and 10 lived in a nursing home. In this sample, 10 agreed to be interviewed. The others denied due to lack of strength or deteriorating health.

**Table 2.** Inclusion and exclusion criteria for older persons and their relatives.

Older persons	
Inclusion criteria	Exclusion criteria
Receiving municipal home health care or care in a nursing home ≥65 years old Perceived by CNs as having palliative care needs (no need for formal palliative enrolment) Perceived by CNs as being frail Having diverse diagnoses (e.g. heart disease, chronic obstructive pulmonary disease, kidney failure, cancer or multi-morbidity) Talking and understanding Swedish Being decision-competent	Having dementia Having severe cognitive problems
**Relatives**	
**Inclusion criteria**	**Exclusion criteria**
Related to an older person who had received the DCI-SWE Talking and understanding Swedish Being decision-competent	Having dementia Having severe cognitive problems

CN = community nurse; DCI-SWE = Swedish Dignity Care Intervention.

Further, the researcher or CN asked the older persons for permission to contact their relatives, and if they agreed, the relative was contacted by telephone for information about the study. Eight relatives were invited to the study and all accepted, eligibility criteria (Table 2).

### Data collection

Data were collected by using questionnaires and individual interviews (Data collection overview, Table 1).

### Demographic data

Demographic data were collected from the participants during interviews (Table 3).

**Table 3.** Older persons’ and their relatives’ socio-demographic characteristics.

Older persons	(N = 18)
**Age**, years, mean (SD)	88 (6.8)
min; max	74; 98
Median	88
**Female, *n* (%)**	13 (72)
**Marital/social status, *n* (%)**	
Married/living with partner	2 (11.1)
Widowed/living alone	16 (88.9)
Type of health care, *n* (%)	
Home care	7 (38.9)
Nursing home	11 (61.1)
**Relatives**	**(N = 8)**
**Age**, years, mean (SD)	68 (10.8)
min; max	47; 83
Median	69
**Female, *n* (%)**	6 (75)
Relationship, *n* (%)	
Wife/husband	1 (12.5)
Sibling	2 (25.0)
Daughter	2 (25.0)
Son	2 (25.0)
Friend	1 (12.5)

SD = standard deviation.

### Questionnaires

Older persons’ dignity-related distress was measured with the PDI,^18^ a 26-item questionnaire scored on a 5-point Likert scale ranging from 1 (not a problem) to 5 (an overwhelming problem). Min score 26 points max 130 points. The international version has demonstrated good internal consistency (α=0.93).^19^ Older persons’ quality of life was measured with the multidimensional McGill Quality of Life-Expanded (MQOL-E), consisting of 22 items scored on a 1–10-point scale.^30,31^ Both questionnaires were conducted at study start (baseline) and at one follow-up (after care actions had been introduced in the older persons’ care) and took 30–60 minutes each to complete. Most older persons filled in their questionnaires by themselves, but some were assisted by a CN/researcher who asked the questions and filled it in. On one occasion, the person recently had been hospitalized and then the CN filled in the questionnaire. These occurrents were marked ‘by proxy’.

### Individual interviews

Ten older persons participated in interviews. A semi-structured interview guide was developed focused on questions regarding experiences of dignity and DCC when using the DCI-SWE. The interviews were conducted face to face (*n* = 5) in the older persons’ nursing home apartment or own home, or, because of the pandemic COVID-19, by telephone (*n* = 5) (M=36 minutes; range 22–55 minutes). Eight relatives participated in interviews, either in the relative’s home (*n* = 3) or at their workplace (*n* = 1) or by telephone (*n* = 4) (M =37 minutes; range 23–60 minutes). All interviews were conducted by the same researcher (A.S.) and were audio recorded, and later transcribed by a professional transcriber. In one case, a relative was uncomfortable with being recorded, and therefore the researcher took notes instead.

### Data analysis

#### Statistical analysis

In the analyses of the questionnaires, non-parametric tests were used. Differences between the two assessment points (baseline and follow-up) were analysed with the Wilcoxon signed rank test.^32^ The PDIs from one older person contained estimates between two values (in 15 items). This was handled by alternately increasing and decreasing to the nearest value.^33^ All analyses were performed using IBM SPSS Statistics, version 26 (IBM Corp., Armonk, NY, USA). *p*-values (two-tailed) <0.05 were considered statistically significant.

### Thematic analysis

Thematic inductive analysis^34^ was utilized to give voice to participants’ experiences of dignity and DCC when using the DCI-SWE (analysis process, Table 4). In phase one, A.S. read all interviews (data corpus) for familiarization and noted initial ideas about patterns. Interviews (data items) were put into the data N-Vivo 12 program (QSR International, Doncaster, Australia), which was used throughout the analysis. In phase two, features (data extracts) from interviews were coded systematically in relation to the study aim and put into different codes (A.S.) that were further discussed within the whole research group. In phase three, the relation between codes and how they could be combined into initial semantic subthemes, was considered (A.S., C.W.H.). In phase four, initial subthemes were reviewed by all researchers to identify if they needed refinement in relation to the coded extracts or to the whole data set. During analysis of the subthemes, initial overarching latent themes were formulated (A.S.). Phase five focused on clarifying, defining and naming the themes based on their true essence (all researchers). A thematic map was developed to get an overview (Table 5). Phase six emphasized writing a narrative of the themes and selecting quotes to increase the study’s credibility.

**Table 4.** Examples of the analysis of qualitative data.

Data extract	Code	Subtheme	Theme
*Interview, older person*			
It was difficult to describe, I have to say. For me, it’s like … how to actually treat other persons and how to experience them and try to be as friendly as possible to everyone really. Yes, something like that, I think, can be dignity	Difficult to describe dignity	Perceptions of the dignity concept	Dignity through life – how to relate to dignity
*Interview, relative*			
Is there anything specific in care that you feel has an impact on the older person’s dignity? They [HCPs] should listen more, that’s … respect as well. Respect, that it is about persons. Listen to them [the older persons], listen to what they want as well. That’s what … I think it is missing much in health care	Listening and showing respect are missing	Being acknowledged by health care professionals	Valuing the individual person in a caring meeting

**Table 5.** Themes and subthemes from qualitative interviews with older persons and their relatives.

Dignity through life – how to relate to dignity	Valuing the individual person in a caring meeting
Perceptions of the dignity concept	Being acknowledged by health care professionals
Maintaining health, abilities and social roles	A pleasant care atmosphere and meaningful activities
The importance of acceptance and of mental strength	Care competence, continuity and resources
About the possibility of discussing dignity aspects	

### Ethical considerations

The study was carried out in accordance with the Declaration of Helsinki.^35^ Written ethical approval was obtained from the Regional Ethical Review Board in Uppsala, Sweden (Reg No. 2014/312), and later by the National Swedish Ethical Review Authority (Reg. No. Ö 10-2019). The municipality’s head of health care gave written permission for the study. Informed consent was collected from CNs, older persons and their relatives both orally and in writing. They were informed that their participation would be voluntary and that they could drop out of the study at any time without explanations. Researchers strived to respect older persons’ vulnerability during all meetings for example by asking them how they wanted the interview situation to be. The researcher performing the interviews had no professional relation to the participants, which meant that the older persons’ healthcare could not be affected by their answers. The participants identities were coded to not be revealed and data material were stored in a locked cabinet.

## Results

Eighteen older persons and eight relatives were included in the study (participant demographics, Table 3). For CNs demographics, see the process evaluation of the DCI project.

### Dignity-related distress

The older persons’ dignity-related distress did not significantly change over time, *p* = 0.44 (Table 6). ‘By proxy measurements’ were one out of 18 at baseline and four out of 17 measurements at the follow-up.

**Table 6.** Baseline and follow-up scores on the Swedish Patient Dignity Inventory (PDI), and magnitude of differences over time.

Questionnaire/item The PDI	N	Medians (interquartile ranges 25^th^–75^th^ percentiles), baseline (T^0^	Range	N	Medians (interquartile ranges 25^th^–75^th^ percentiles), follow-up (T^1^) (after care actions were introduced in care)	Range	Difference (T^1^)–(T^0^) mean score	*P*-value^a^	Items rated ≥3 at T^0^ (*n* = %)	Items rated ≥3 at T^1^ (*n* = , %
1. Experiencing physically distressing symptoms (such as pain, shortness of breath, nausea)	18	**3.0† (2.00–4.00)**	1–4	16	**3.0† (2.00-4.00)**	1–5	–	0.59	**67%**	**63**
2. Feeling depressed	18	2.0 (1.00–2.25)	1–4	17	2.0 (1.00–2.50)	1–4	–	1.00	22%	24
3. Feeling anxious	18	2.0 (1.00–2.00)	1–4	17	2.0 (1.50–3.00)	1–4	**0.5**	0.09	11%	29
4. Feeling uncertain about my illness and treatment	18	1.5 (1.00–3.00)	1–4	17	2.0 (1.00–3.00)	1–3	0.2	0.33	39%	41
5. Worrying about my future	18	1.5 (1.00–2.25)	1–4	17	2.0 (1.00–2.00)	1–4	0.1	0.59	22%	29
6. Not being able to carry out tasks associated with daily living (e.g. washing myself, getting dressed)	18	2.0 (1.00–3.00)	1–5	17	2.0 (1.00–3.00)	1–4	–	0.59	33%	29
7. Not being able to attend to my bodily functions independently (e.g. needing assistance with toileting-related activities)	18	2.0 (1.00–3.00)	1–5	17	2.0 (1.00–3.00)	1–4	0.1	0.56	39%	24
8. Not being able to think clearly	18	1.0 (1.00–2.00)	1–4	17	1.0 (1.00–2.00)	1–3	−0.2	0.38	17%	6
9. Feeling that how I look to others has changed significantly	17	1.0 (1.00–2.50)	1–4	17	1.0 (1.00–2.00)	1–4	–	0.74	24%	18
10. Feeling like I am no longer who I was	18	3.0 (1.00–3.00)	1–4	17	3.0 (2.00–4.00)	1–4	0.3	0.13	**56%**	**53**
11. Not feeling worthwhile or valued	18	1.0 (1.00–2.00)	1–4	17	1.0 (1.00–2.00)	1–4	–	1.00	11%	18
12. Not being able to carry out important roles (in relation to family, friends or society)	18	2.0 (1.00–2.25)	1–4	17	1.0 (1.00–2.50)	1–4	–	0.53	22%	24
13. Feeling that life no longer has meaning or purpose	18	1.0 (1.00–2.00)	1–3	16	1.0 (1.00–2.75)	1–4	0.3	0.10	11%	25
14. Feeling like I have not made a meaningful and lasting contribution during my lifetime	18	**1.0†† (1.00–1.25)**	1–2	17	1.0 (1.00–1.00)	1–2	–	0.56	**0%**	**0**
15. Feeling I have ‘unfinished business’ (e.g. things left unsaid or incomplete)	18	1.0 (1.00–2.00)	1–3	17	1.0 (1.00–1.50)	1–3	−0.1	0.16	6%	6
16. Feeling that I don’t have control over my life	18	1.0 (1.00–3.00)	1–3	17	2.0 (1.00–3.00)	1–4	0.2	0.10	33%	29
17. Not being able to accept the way things are	18	2.0 (1.00–3.00)	1–3	17	1.0 (1.00–2.50)	1–3	−0.1	0.79	28%	24
18. Feeling that I am no longer able to mentally ‘fight’ the challenges of my illness	18	1.5 (1.00–2.25)	1–4	17	2.0 (1.00–3.00)	1–4	0.3	0.16	22%	29
19. Not being able to continue with my usual routines	18	2.0 (1.00–3.00)	1–4	17	2.0 (1.00–2.50)	1–4	**−0.3**	0.38	39%	24
20. Concern that my spiritual life is not meaningful	17	1.0 (1.00–1.50)	1–3	17	1.0 (1.00–1.00)	1–3	−0.1	0.32	6%	6
21. Feeling that my illness and care needs have reduced my privacy	18	1.0 (1.00–2.00)	1–4	17	1.0 (1.00–2.00)	1–5	0.3	0.40	6%	12
22. Not feeling supported by my community of friends and family	18	**1.0†† (1.00–1.00)**	1–3	17	**1.0†† (1.00–1.00)**	1–1	−0.2	0.10	6%	**0**
23. Not feeling supported by my health care providers	17	1.0 (1.00–1.00)	1–4	17	1.0 (1.00–1.00)	1–3	−0.1	0.56	6%	6
24. Not being treated with respect or understanding by others	18	1.0 (1.00–2.00)	1–3	17	1.0 (1.00–2.00)	1–3	–	1.00	6%	6
25. Feeling that I am a burden to others	18	2.0 (1.00–2.25)	1–4	17	2.0 (1.00–2.50)	1–4	–	0.74	22%	24
26. Concerned about how my death will affect my family and friends	18	1.0 (1.00–2.25)	1–3	17	1.0 (1.00–2.00)	1–4	–	0.74	22%	18
Sum of all ranks	16	46.0 (33.25–52.75)	29–76	15	45.0 (37.00–60.00)	28–65	2.00	0.44		

Frequency: 1 = not a problem; 2 = a slight problem; 3 = a problem; 4 = a major problem; 5 = an overwhelming problem (higher scores indicating greater dignity-related distress);

^a^Wilcoxon signed rank test; †the highest and ††the lowest medians in rated PDI items at T^0^ and T^1^; the largest and smallest difference since T^0^ are marked in bold.

### Quality of life

The older persons’ overall quality of life did not significantly change over time (*p* = .64). Only in the psychological dimension was the decrease statistically significant (*p* = .01) (Table 7). By proxy measurements were one out of eighteen at baseline and 7 out of 17 at follow-up.

**Table 7.** Baseline and follow-up scores on the Swedish McGill Quality of Life-Expanded (MQOL-E) questionnaire, and differences over time.

Questionnaire/dimension MQOL-E	*N* (T^0^)	Medians (interquartile ranges 25^th^-75^th^ percentiles), baseline (T^0^)	*N* (T^1^)	Medians (interquartile ranges 25^th^-75^th^ percentiles), follow-up (T^1^)	Difference (T^1^)–(T^0^), mean score	*P*-value^a^
Overall quality of life	18	6.0 (4.75–7.00)	17	5.0 (3.50–8.00)	−0.2	0.64
Physical dimension	18	**5.3†† (4.33–7.08)**	17	**4.7†† (3.00–6.17**)	−1.4	0.08
Psychological dimension	18	7.9 (5.75–10.00)	17	7.3 (2.25–8.88)	**−1.7**	**0.01**
Existential dimension	18	7.1 (5.44–9.50)	17	8.3 (5.00–9.00)	−0.4	0.38
Social dimension	18	8.8 (7.17–10.00)	17	8.0 (6.83–9.67)	−0.5	0.46
Burden	18	10.0 (7.25–10.00)	17	8.0 (4.50–10.00)	−1.4	0.12
Environment	18	8.0 (7.00–10.00)	17	9.0 (7.50–10.00)	**0.5**	0.98
Cognition	18	7.8 (6.00–10.00)	17	8.0 (6.75–10.00)	0.2	0.26
Health care	18	9.2 (7.92–10.00)	17	8.3 (7.33–9.33)	−0.3	0.19
Finance	18	**10.0† (8.00–10.00)**	17	**10.0† (9.00–10.00)**	0.3	0.89
Total	18	7.7 (6.97–8.82)	17	7.6 (6.04–8.80)	−0.5	0.16

MQOL-E items ranked from 0 to 10 (the higher the estimate, the better the quality of life); †the highest and ††the lowest medians rated in MQOL-E items at T^0^ and T^1^; the largest and smallest difference since T^0^ are marked in bold;

^a^Wilcoxon signed rank test.

### Responses about dignity and dignity-conserving care

Older persons’ and relatives’ experiences comprised two themes: ‘Dignity through life – how to relate to dignity’ and ‘Valuing the individual person in a caring meeting’ and seven subthemes (Table 5). Unless otherwise explained in the text, CNs are included in the term ‘HCPs’.

### Dignity through life – how to relate to dignity

Most older persons’ dignity changed throughout life, and there were aspects that had a role in how they experienced their dignity.

### Perceptions of the dignity concept

The dignity concept was unfamiliar to some older persons and their relatives. It was stated that dignity was something health care did not work much with or did reflect much on. Even if some had uncertainties about the concept many described what was important for their dignity. The older persons linked dignity to being understood, respected and being friendly. The relatives linked dignity to showing love and consideration, to being a human and the importance of communicating.

*… that dignity is maintained even if you end up in a nursing home or need other services. […] that everything is done for the good of the individual.* (Relative 1)

Some expressed more a clear view of dignity. One older person said that when people accept your opinion, that is a kind of dignity; and one relative said that every person has a unique value, and that dignity cannot really be taken away.

### Maintaining health, abilities and social roles

Some older persons expressed happiness for having good health despite their age, which helped them experience dignity. Others linked dignity to their recovery and dependence on help. Together with relatives, they described that older persons’ dignity could be affected by changed conditions leading to limitations to do things. Some older persons described the will to continue doing what they could. Both older persons and relatives highlighted that older persons’ ability to speak for themselves and continue with social roles could help maintain their dignity. Older persons described the lack of a meaningful role, and how they strove to maintain their dignity while meeting others.

*I don’t think I have any [dignity] […] But if I say that I experience you as a guest or acquaintance sitting here, then I would like to try to make the best of what I can offer.* (Older person 1)

Relatives supported older persons’ social roles and viewed their ability to live their life as they used to as a part of their dignity. They thought creating relationships with HCPs could help some maintain their dignity.

### The importance of acceptance and of mental strength

Accepting one’s situation and being accepted by others was important for older persons’ dignity. Some looked back at their lives and realized they had done well, and they felt dignity despite bodily concerns or loneliness. When asked about current dignity, an older person said:

*You can say it like this, you live a little differently, you have it a little different, but you must accept it.* (Older person 2)

Relatives thought it was a process for older persons to accept life changes. From both perspectives, it was described how older persons’ mental strength influenced their dignity, but that this could vary depending on the situation.

### About the possibility of discussing dignity aspects

For older persons and relatives, it was important to put dignity and communication on the agenda and they described the DCI-SWE as raising older persons’ quality of life. Some older persons did not know what could be done for their dignity and hoped the DCI-SWE would become useful. Others thought the DCI-SWE was a way to ventilate their thoughts and resolve concerns. Completing the PDI required concentration, and both the older persons and the relatives expressed that some questions were strange or difficult. Still, they felt that completing the PDI was relevant and served as a reminder of important things. Most older persons had confidence in their CN and felt secure to have DCI conversations. Some older persons became closer to their CN and described that they had been listened to. According to relatives, the DCI-SWE allowed CNs to follow an older person over time, and to include older persons opinions in the care planning. Some relatives lacked a context for discussing end-of-life issues and suggested meetings with the CN should be held every 3 months. Most relatives did not participate in DCI conversations, but they gave examples of changes in the older persons’ care that had occurred after the conversations (as including nutritional beverages). When asked whether the DCI-SWE was helpful, one relative said,

*…it depends on the staff as well. What staff [it is] ...the way the staff work.* (Relative 2)

### Valuing the individual person in a caring meeting

Older persons and their relatives described the importance of showing older persons they are valuable.

### Being acknowledged by health care professionals

Both the older persons and their relatives expressed the importance of older persons being seen and that relationships were important. Undignified treatment could demolish older persons’ quality of life and impact their dignity. Some older persons had experienced unworthy treatment. Older persons expressed that DCC was about HCPs showing that they want to help, being sensitive to needs and knowing the older person by name. Their dignity was affected by which staff member was on duty. They generally wished for the regular staff because they were known to them, thus giving opportunities for improved care. Additionally, some said, it was difficult to get in touch with some HCPs. A close relationship was difficult to create if the HCP came too rarely. To enable conversations, HCPs had to show interest:

*… if someone doesn’t seem to show interest, then she [the older person] doesn’t show anything back. I think that’s her dignity, right?* (Relative 3)

Both the older persons and their relatives expressed the value of being heard and respected. It felt humiliating not being understood. Most of them valued their care, but some had experiences of HCPs not taking the time to talk, or only talking to each other. The relatives said it was difficult for older persons to keep up and feel worthy if they did not understand HCPs’ language or had problems with hearing or seeing. They suggested that activities could help older persons to be seen by others, but that some older persons wanted someone to talk to rather than do an activity. Some relatives expressed that HCPs often listened, and that the care was based on older persons’ needs.

### A pleasant care atmosphere and meaningful activities

A pleasant care atmosphere was relevant for achieving DCC. Older persons appreciated a good tone of voice, humour and compassionate staff to experience dignity. They were aware that not all older persons were as well off as themselves. It was tough for them to see others in worse health or hear about, someone who had died. According to the relatives, DCC was about creating a warm atmosphere and supporting older persons in all possible ways. They felt assured if they knew that the older person was in good hands. Some thought it affected older persons’ dignity to live in a nursing home. One relative described an earlier experience from a short-term accommodation:

*She didn’t feel treated with dignity. […] It was more like being in storage to be there.* (Relative 4)

The relatives spoke with appreciation of the activities introduced after the DCI-SWE, like meeting with the care dog or an outdoor activity. However, older persons talked about activities they could not do anymore, that some wishes would just remain a dream. Some older persons thought every day is like the next.

### Care competence, continuity and resources

Care competence was highlighted by all as important for DCC. The relatives expressed that knowledge about dignity care was sometimes lacking and that care staff needed to reflect on human dignity. They stated that HCPs must be able to communicate with older persons, and that they wanted the older persons’ last phase in life to turn out well. Most older persons expected HCPs to have knowhow and were concerned when HCPs were inexperienced and did not know about their preferences and needs.

*I shouldn’t have to have more knowledge than people working in health care.* (Older person 3)

Most older persons had no experiences of undignified care. Older persons and relatives underlined that HCPs did their best, and that older persons’ quality of life increased when they got the right kind of help. They also emphasized care continuity. Daily visits enhanced older persons’ quality of life, and not meeting so many different HCPs was important for their dignity. Although most relatives thought the continuity was generally good, they said that new or unknown HCPs just performed their tasks, making it difficult for the older person to start a conversation. All emphasized that health care ought to have adequate resources to achieve DCC. The older persons related that, when the regular staff (home care) were on leave the help could become scant. Further, HCPs’ ability to listen to them often had to do with staff availability, and HCPs’ limited time was a barrier to solving concerns. Likewise, the relatives expressed that a high staff turnover and limited time impacted older persons’ dignity. They wanted CNs to participate more in care to enhance the quality.

## Discussion

The findings suggest that the DCI-SWE has the potential to be a forum for older persons to talk about personal aspects and dignity issues. It can clarify what support older persons want, to maintain their personality. For example, the DCI-SWE can give openings for older persons to talk about ‘no longer being the person I was’, which many of them estimated as a problem in the PDI. This issue has also been reported to be predominant for older persons in other studies,^36,37,38^ since self-image and identity are key factors in promoting dignity.^39^

Showing older persons that they are valuable were seen to underpin their dignity. The relationship with HCPs was highlighted by both the older persons and their relatives. Being seen and heard was important for older persons’ dignity. The DCI-SWE helped some older persons become closer to the CN that used the intervention, which is consistent with an earlier study by Johnston and colleagues.^40^

In previous research, Chochinov et al.^41^ findings showed that, older persons felt less supported by family and friends compared to other groups. Outcomes in our study indicated that older persons felt social support from family, friends and HCPs. By maintaining social roles, older persons’ dignity can be supported, and the HCPs were helpful in supporting this. To preserve older persons’ dignity, multidimensional policies are needed, where older persons are involved in decisions about their care.^42^ The interviewed relatives suggested meeting with the CN every 3 months, similar as suggested in a previous study by Johnston and colleagues.^15^ With regular DCI conversations, knowledge of older persons’ preferences may be improved.

To conserve older persons’ dignity, maintaining health and abilities was important, and this was both mentioned by the participants, and shown in the older persons’ ratings of physical distress (which had the highest median of all the items in the PDI) and ratings of the physical dimension of quality of life (which had the lowest median in the MQOL-E), although non-significant. Similar findings were seen in Chochinov et al.^41^ where older persons reported physical distress as a problem, confirming that it is a prevalent problem impacting dignity.

Moreover, the older persons’ psychological quality of life decreased over time (*p* = .01). This could possibly be explained by that, with CNs’ use of the DCI-SWE, older persons became more aware of their psychological wellbeing. Therefore, care actions related to the psychological dimension may require more attention. Those who care for older persons on an everyday basis need to talk about psychosocial issues.^43^ Basic counselling skills training has been recommended for HCPs to develop adequate competency and give attention to a person’s holistic needs.^44^

In this study, the older persons and relatives emphasized care competence, continuity and resources, which indicate that DCC is complex work. Although older persons have complex needs,^45^ the goal must always be to deliver high quality of care.^42^ However, the responsibility for quality in care cannot be placed solely on nurses as they have multiple demands, and little formal power.^45^ Therefore, politicians need to prioritize resources to older persons’ care, and health care leaders must show what care pathways are important. Compassion-driven health care has been argued to safeguard dignity at end of life,^44^ and policy makers and managers can help nurses obtain resources to ensure that older persons can die with dignity.^45^ Using the DCI-SWE can help establish DCC as a natural part of health care. Therefore, further research on the DCI-SWE can benefit older persons and facilitate challenging times within municipal health care.

## Methodological considerations

The TREND checklist was used for methodological considerations.^46^ Our study contributed to knowledge about the context, the target population and the outcome instruments. The COVID-19 pandemic affected the municipal health care during the study period, which impacted the sample sizes and the amount of data collected.

In research involving older persons, it is beneficial using mixed methods to ensure both that their voices will be heard and that prevalent problems will be identified. However, because of the small sample size our findings must be interpreted with caution as they may be due to chance, and therefore must be evaluated by drawing on supporting evidence. The low number of older persons may be explained by that only a small percentage of persons suitable to take part in the DCI-SWE live in nursing homes. The proportion of older persons moving to nursing homes and die shortly after has increased,^47^ therefore many older persons may have been too frail to participate. With advancing age, participation in research normally declines.^48^ Thus, a larger focus on home care in future studies may be suitable. Non-randomized designs can be used to study rare adverse events,^24^ but a randomized controlled trial can help generalize the outcomes of the DCI-SWE. With adjustment for confounders, the reliability could increase.

There was likelihood of selection bias^46^ as participants were not randomized. More older women than men participated. However, a natural cause could have been that in one nursing home 73% and in the other nursing home, 67% of residents were women. Therefore, the sex ratio in our study population may be related to women living longer than men^49^ or related to gender issues. Previous studies have shown that participation in research activities related to sex can be mixed.^50^

To evaluate an intervention properly, a crucial aspect is the choice of outcome measures.^12^ Since our study used the PDI, which is also included in the DCI-SWE, there was a clear risk of bias. The researchers were aware, but there was no other validated dignity instrument translated at this time in Sweden.

The qualitative part of the study was a way to capture the human experiences by letting older persons and relatives describe these experiences.^51^ The credibility in the study was enhanced as both the perspectives of older persons and relatives were involved. Quotations were used to illustrate the findings, something that also enhanced the confirmability. Transferability is difficult to accomplish in qualitative studies as the result must be viewed and understood according to the specific context and geographical area. However, the study methods and the participants have been described as clearly as possible, along with providing detailed descriptions of the study context. Thereby the readers are given the possibility to determine themselves how far the transferring of the results can be made. By using the same interview guide in the interviews, letting the same researcher perform all interviews, and discuss and check the relevance of themes and subthemes in the study, dependability has been enhanced.^52^

## Conclusions

The DCI-SWE provides a forum to talk about dignity issues and a way to work with communication in care. Creating a relationship between the older person and the CN using the DCI-SWE is crucial if the intervention is to be beneficial. According to the older persons and their relatives, to maintain older persons’ dignity it is important to maintain their health, abilities and social roles, and for them to have a mental strength. Likewise, acceptance, both from others and from oneself is vital. Older persons need to feel valued within care; therefore, there must be a pleasant care atmosphere and meaningful activities. Older persons’ psychological quality of life often decreases due to low autonomy related to few opportunities to engage in everyday activities, being unable to do things they used to do, and not feeling in control of their future.^53^ This signals that psychological care actions require attention. With communications training for CNs, older persons’ psychological wellbeing may increase. As older persons’ dignity-related distress increased slightly but non-significantly, the effectiveness of the DCI-SWE must be determined in a larger study. Conclusively, care competence, continuity and resources need to be established if care organizations are to achieve DCC.
